# Anisotropic Behavior in Microstructures and Properties of Refractory Tungsten Metal Produced by Laser Powder Bed Fusion

**DOI:** 10.3390/ma18163910

**Published:** 2025-08-21

**Authors:** Jinguo Ge, Heming Wu, Hongsen Liu, Yanan Zhu, Yan Chen, Wangwei Zhan, Liang Zhang, Zhuming Liu

**Affiliations:** 1 Institute of Semiconductors, Guangdong Academy of Sciences, Guangzhou 510650, China; 2 Department of Mechanical and Manufacturing Engineering, Trinity College Dublin, The University of Dublin, Parsons Building, D02 PN40 Dublin, Ireland; 3 School of Mechanical and Electrical Engineering, Guilin University of Electronic Technology, Guilin 541010, China; 4 Institute of Intelligent Manufacturing Technology, Shenzhen Polytechnic University, Shenzhen 518055, China

**Keywords:** refractory tungsten metal, laser powder bed fusion, anisotropic behavior, microstructure and crack, hardness and wear properties

## Abstract

This work employed laser powder bed fusion (LPBF) technology to prepare pure tungsten (W) metal components and investigated their internal defects, microstructural characteristics and mechanical properties within the horizontal and vertical planes to evaluate their anisotropic behavior. The steep temperature gradient and extremely rapid cooling rate during the LPBF process caused the as-deposited W grains to grow in a columnar crystal structure along the vertical height direction, with cracks propagating along the high-angle grain boundaries (HAGBs). Although the near-equiaxed W grains within the horizontal plane were finer than the epitaxial grains within the vertical plane, the increased number of cracks within the horizontal plane weakened the fine-grained strengthening effect, resulting in lower hardness and wear resistance within the horizontal plane than within the vertical plane. The wear behavior transformed from a comprehensive wear mechanism involving delamination wear and abrasive wear within the vertical plane to an abrasive wear mechanism with slight adhesive wear within the horizontal plane. The reported results demonstrate that the anisotropic behavior of hardness and wear resistance within the different deposition planes was mainly attributed to the differences in microstructure and crack distribution between the horizontal and vertical planes of LPBF-fabricated W parts.

## 1. Introduction

Tungsten (W) is an important refractory metal with many unique physical and chemical properties, such as high melting point, high density, high strength, good thermal conductivity, and low thermal expansion coefficient [1,2]. It is widely used in aerospace, medical, military, electronics, and nuclear industries [3]. In the nuclear industry, tungsten is the most promising plasma-facing candidate material due to its excellent heat resistance, high sputtering threshold, and low activity under neutron irradiation [4]. However, in fields such as nuclear energy, the W material usually undergoes continuous friction, which leads to material wear and surface damage. This may affect its performance and shorten its service life [5,6]. In the medical field, tungsten is often used to prepare anti-scatter grids because of its high density and superior radiation absorption capabilities [7]. In the semiconductor field, tungsten is selected to manufacture cathodes for high-power emission tubes due to its high melting point, good high-temperature stability, and low work function [8].

However, pure W metal also has some drawbacks, including a high melting point (3410 °C), poor plasticity and toughness, and a high ductile–brittle transition temperature (200–500 °C), making it difficult to fabricate and process using conventional casting or machining methods [9]. Typically, W metal parts are manufactured using powder metallurgy (PM) [10], metal injection molding (MIM) [11], or spark plasma sintering (SPS) [12]. However, as-sintered W metals have several drawbacks, including poor performance, lower relative density, and difficulty in controlling impurity content [13]. Furthermore, the production of some W components involves complex features like curved surfaces, micropores, and grooves, which are challenging to manufacture using traditional PM techniques and machining [14].

In recent years, additive manufacturing technology, particularly laser powder bed fusion (LPBF), has opened up a new manufacturing route for W parts [15]. LPBF technology adopts powder as a raw material and a high-energy laser beam as a heat source to produce high-quality precision metallic components under the protection of high-purity argon gas [16]. It can resolve many issues that cannot be resolved during W metal preparation using traditional processes, allowing it to fully exploit its advantages in shaping complex components with reasonable deposition quality [17]. Furthermore, due to the rapid heating and cooling characteristics of additive manufacturing, the resulting microstructure is fine (with some grains smaller than 1 μm) [18]. Compared to traditional preparation methods, such as PM, the LPBF-fabricated parts commonly have higher hardness and density, showing promising application prospects [14,17].

In contrast to traditional PM processes, the periodic reciprocating motion of the laser heat source during the LPBF process results in a strong thermal–mechanical interaction with the surrounding environment, and the thermal boundary of the molten pool exhibits nonlinear time-varying characteristics under long-term heat sink conditions [19,20]. This results in extreme complexity and variability of the non-equilibrium microstructure at different deposition micro-regions of the LPBF-fabricated parts, manifesting as porosity and incomplete fusion defects, molten pool boundaries, elongated columnar and equiaxed grains, cellular and elongated subgrain structures, etc. [20,21]. Moreover, these microstructures, which determine the mechanical properties, are highly dependent on the thermal history (in situ solidification/micro-heat treatment effects) during the LPBF process [22]. Porosity defects and inclusions are frequently found at the interfaces between adjacent layers, which have a negative impact on the material’s structural uniformity and are a major cause of the anisotropy behavior in mechanical properties [23]. Additionally, unlike the traditional processes, the layer-by-layer stacking characteristic of LPBF technology causes different deposition planes to exhibit different molten pool morphological features [24]. Molten pool boundaries parallel to the vertical direction resemble fish scales, while those perpendicular to the deposition direction are pipe-like [25]. It is well known that molten pool boundaries are weak positions in terms of mechanical properties, so different molten pool morphologies could be one of the reasons for the anisotropy in mechanical properties [25]. Besides these two factors, the most important causes of anisotropy are differences in grain size, grain morphology, and the aspect ratio of grains in different stress directions [26]. According to the grain boundary strengthening theory, the strength of metallic materials is inversely proportional to the grain size, and high-angle grain boundaries (HAGBs) can effectively hinder dislocation movement [27]. During the plastic deformation process, dislocations cross different numbers of grain boundaries in different directions, resulting in mechanical properties that differ depending on the stress direction [28].

Typically, the microstructure of LPBF-fabricated metallic components consists primarily of elongated and equiaxed grains. Elongated grains parallel to the deposition direction produce mechanical anisotropy, while nearly equiaxed grains and a mixture of elongated–equiaxed grains can reduce or even eliminate this anisotropic behavior of mechanical properties [29]. Because of the extremely fast cooling rate (>10^3^ °C/s) during the LPBF process, epitaxial grains are easily formed, which ultimately leads to mechanical anisotropy in different deposition orientations [30]. According to previous studies, the phenomenon of mechanical anisotropy is common in LPBF-fabricated parts made of stainless steel [31], titanium alloys [32], aluminum alloys [33], and NiTi shape memory alloys [34]. Despite numerous studies on the internal defects, microstructures, and overall properties of the pure W parts produced by LPBF, reports on the anisotropy of their microstructures and properties are scarce.

Given the current relative lack of research on the anisotropy of LPBF pure W parts, this study focuses on the microstructure evolution and performance differences in these components under different deposition directions. Specifically, through a systematic comparative analysis of the microstructure, Vickers hardness, and wear resistance of the tungsten components prepared by LPBF in the horizontal (X-Y) and vertical (X-Z) directions, and in comparison with traditional tungsten matrix materials, the anisotropic behavior and formation mechanism of these components are deeply revealed, providing theoretical support and technical references for achieving the regulation of their microstructure and performance optimization.

## 2. Materials and Methods

### 2.1. Feedstock and Manufacturing

**Figure 1:**
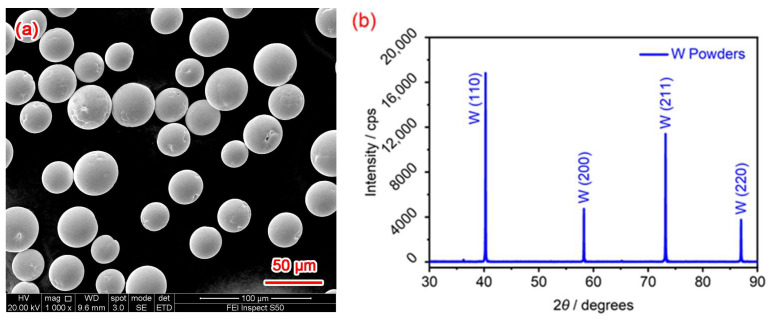
Characterization of the pure tungsten powders: (**a**) SEM morphology and (**b**) XRD pattern.

The raw material adopted in this work was pure W powders, which were fabricated by electrode induction melting gas atomization (EIGA) technology and were sourced from Jiangsu Vilory Co., Ltd. (Xuzhou, China). As shown in Figure 1, W powder particles were spherical or nearly spherical, with a body-centered cubic (B.C.C.) lattice structure. The nominal particle size was in the range of 15 μm to 53 μm (D10: 19.8 μm, D50: 29.3 μm, D90: 40.7 μm). According to the quality certificate provided by the supplier, the chemical composition and oxygen content of this powder comply with the relevant Chinese national standards. The concentration of impurities provided by the supplier was 170 ppm, <5 ppm, 5 ppm, and 5 ppm for oxygen, nitrogen, sulfur, and phosphorus, respectively. Before LPBF, the as-received W powers were dried in a vacuum oven at 150 °C for 4 h.

The solid W parts were manufactured on the pure W substrate using commercial LPBF equipment (M290, EOS GmbH, Krailling, Germany), which is equipped with a Yb-fiber laser (wavelength: 1064 nm, maximum output: 400 W) having a spot size of 100 μm. A systematic optimization of LPBF parameters for pure tungsten was conducted by adjusting laser power (150–350 W), scanning speed (100–600 mm/s), and hatch spacing (0.06–0.1 mm). The optimized LPBF processing parameters were as follows: laser powder of 360 W, scanning speed of 200 mm/s, layer thickness of 30 μm, and hatch distance of 80 μm. Considering the extremely high oxidation sensitivity of W powder in a high-temperature environment, high-purity inert argon was continuously supplied into the air-tight chamber to ensure an oxygen concentration of less than 0.1 vol.%. In addition, the alternate hatch-patterned scanning strategy was used to reduce the internal residual stress during sample deposition, where the laser scanning direction between adjacent layers was set as 67°.

### 2.2. Microstructure Characterization

Cubic samples measuring 5 × 5 × 5 mm^3^ were fabricated for analysis of deposition quality and microstructural characteristics. Three-dimensional (3D) surface morphologies of the LPBF-fabricated W samples were observed by laser scanning confocal microscopy (LSCM, Olympus OLS4100, Olympus Corporation, Tokyo, Japan). The arithmetical mean deviation of the profile (namely, *Ra* value) was extracted from the 3D surface morphology to evaluate the surface quality. Measurements were conducted on three samples with consistent results; representative data from one sample is presented. It is defined as the arithmetic mean deviation of the surface peaks and valleys along the measuring line. The lines are perpendicular to the tracks and along the vertical height direction on the top and side surfaces, respectively. Each sample was measured on one top surface and one side surface. For each surface, a single line scan was performed at a fixed central position, with a line length corresponding to the field of view distance of 2578 μm. After mounting, the specimen was sequentially ground with 400#, 800#, 1200#, and 2000# abrasive papers, followed by polishing with 0.1 μm diamond paste. It was then ultrasonically cleaned in 75% alcohol for 5 min. To investigate the relationship between grain boundaries and crack paths, we define the plane perpendicular to the deposition direction as the X-Y plane and the plane parallel to the deposition direction as the X-Z plane. The polished cross-sections of LPBF-fabricated W samples within the X-Y and X-Z planes were etched in a solvent (25–28 wt.% ammonia: 35 wt.% hydrogen peroxide = 1:3, in volume) and then observed by LSCM.

The phase constitutes of W powders, the LPBF-fabricated parts, and the corresponding base metal and it was examined by an X-ray diffractometer (XRD, Rigaku SmartLab, Tokyo, Japan). The range of the diffraction angle (2θ) was 30–90° and the scanning speed was 5°/min at a step of 0.02°. Following the standard metallographic procedures, the microstructural samples were further polished on a VibroMet^®^ 2 (Buehler Ltd., Lake Bluff, IL, USA) vibration polishing machine for 5.5 h to remove any surface residual stress layers. Electron backscatter diffraction (EBSD, Symmetry S3, Oxford Instruments, Abingdon, UK) technology was adopted to characterize texture within the X-Y and X-Z deposition planes, and the data was analyzed using KHL Channel 5 software.

### 2.3. Hardness and Wear Testing

**Figure 2:**
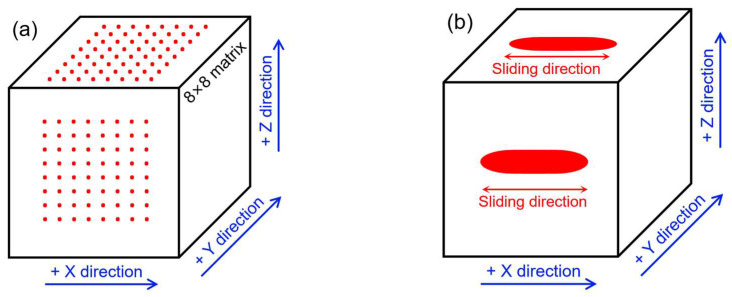
Schematic illustration of hardness (**a**) and wear (**b**) testing for the LPBF-fabricated W parts.

As schematically depicted in Figure 2a, the hardness mapping distribution was measured by a digital micro Vicker hardness tester (HVS-1000A, Laizhou Weiyi Testing Instrument Manufacturing Co., Ltd., Laizhou, China) within the X-Z and X-Y deposition planes, where the Z-direction denotes the vertical height direction and the X-Z and X-Y planes denote the vertical and horizontal planes of the LPBF-fabricated W parts, respectively. The load and indentation time were set as 100 g and 15 s, respectively. Micro-hardness mapping measurements were conducted at eight equidistant positions using an array of 8 × 8. The distance between the adjacent indentation positions was 500 μm. The measurements were performed on three samples, which yielded similar results, and the data from one of them was presented. To serve as a reference, commercially cast pure tungsten was used as the base metal for comparison, for which the hardness was measured at seven evenly distributed points.

The wear properties of LPBF-fabricated W parts within the different deposition planes were tested using a CFT-1 type material surface performance tester, as schematically shown in Figure 2b. Before the experiment, the X-Y and X-Z surfaces were polished with 400 grit, 800 grit, 1200 grit, and 2000 grit sandpaper, followed by ultrasonic cleaning and drying to eliminate the original rough surface or wire-cutting scratches. The measurements were performed on three samples, which yielded similar results, and the data from one of them were presented. The wear experimental parameters were as follows: a loading load of 50 N, a wear time of 30 min, a wear distance of 6 mm, and a friction speed of 400 r/min. The upper friction pair was a silicon nitride ball with a diameter of 5 mm, while the lower friction pair was the LPBF-fabricated W parts. After the wear test, the wear scar morphology was examined using LSCM, and the cross-sectional profiles of the wear scars were extracted to investigate wear resistance within different deposition planes and the corresponding W base metal. Scanning electron microscopy (SEM, Inspect S50, FEI, Hillsboro, OR, USA) was also used for the exploration of the wear failure mechanism through the observation of wear tracks on the worn surface.

## 3. Results and Discussion

### 3.1. Deposition Quality

**Figure 3:**
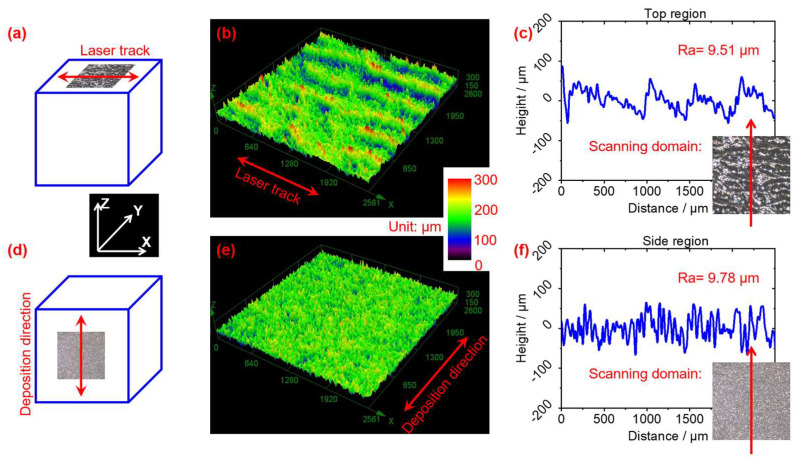
Surface characteristics of the LPBF-fabricated W parts: (**a**–**c**) top surface and (**e**,**f**) side surface; and (**a**,**d**) schematic graph showing the scanning domain, (**b**,**e**) 3D surface, and (**c**,**f**) contour line and *Ra* value.

Figure 3 shows the top and side surface characteristics of LPBF-fabricated W parts obtained using LSCM. The top and side surfaces have achieved excellent deposition quality, with no obvious defects such as pores or cracks. The roughness values (top surface: 9.51 ± 0.31 μm, side surface: 9.78 ± 0.52 μm) are significantly less than the original powder particle size (15–53 μm, as shown in Figure 1a). This indicates that the applied laser energy density was suitable to achieve full melting of the powder, thereby producing relatively smooth surfaces. When the laser beam acted on the W powder with insufficient energy density the short interaction time and low heat input resulted in a smaller molten pool that failed to spread completely before solidification [35], resulting in a rough surface with some unmelted powder adhering to it, thus increasing the surface roughness of the W parts. Conversely, when the laser beam acted on the W powder with excessive energy density, the longer interaction time and higher heat input caused the liquid melt pool to become turbulent due to the intense laser impact. This turbulence led to powder splashing, resulting in a rough deposition surface [36]. At an appropriate energy density, the laser beam effectively melted the W powder, allowing the liquid metal to wet and spread quickly, preventing powder splashing [36], and ultimately achieving a good surface forming quality, as shown in Figure 3.

**Figure 4:**
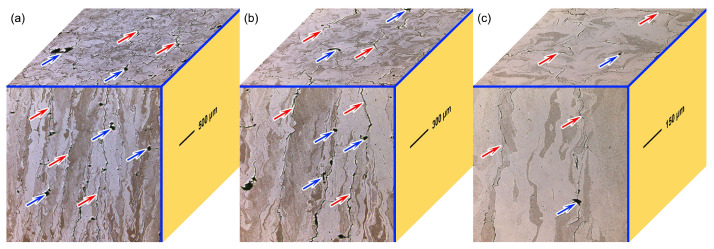
LSCM images of LPBF-fabricated W parts reveal the relationship between grain boundaries and crack propagation paths within the horizontal and vertical deposition planes, (**a**) 5×, (**b**) 10×, (**c**) 20×. (The blue arrows point to voids, and the red arrows point to cracks).

Figure 4 illustrates the LSCM morphology of vertical and horizontal cross-sections of LPBF-fabricated W parts. It is evident that cracks within in the vertical plane are dispersed along the vertical height direction, whereas those within the horizontal plane are arranged in a mesh-like pattern. The microcracks that formed within the LPBF-fabricated pure W parts are primarily due to the thermal contraction. This phenomenon occurred due to two main reasons. Firstly, pure W metal has a higher ductile-to-brittle transition temperature range (200–500 °C), which increases its susceptibility to microcrack initiation and propagation. Secondly, the rapid cooling rates and the substantial temperature gradients generated during the LPBF process also contributed to the formation of microcracks. The significant temperature differences between adjacent layers and the rapid cooling speed resulted in thermal residual stresses. When these residual stresses within the brittle material reached or exceeded the metal’s yield strength, they could trigger the initiation and propagation of cracks. Moreover, tungsten’s inherent metallurgical characteristics, such as low-temperature brittleness and poor fracture toughness, exacerbated the tendency of crack propagation.

When the cracks and grain boundaries within the horizontal and vertical planes in Figure 4 are compared, it is clear that the cracks in pure W parts prepared by LPBF propagate along the grain boundaries. This is primarily due to the sensitivity of the grain boundaries to impurities in the LPBF-fabricated W parts, with many scholars believing that oxygen impurities are the main culprit [19]. During the LPBF process, oxygen is segregated at the grain boundaries in the form of tungsten oxides, weakening the bonding strength of the grain boundaries while also providing preferential sites for crack initiation [37]. Subsequently, the grain boundaries provide longer unrestricted slip paths for crack propagation, resulting in intergranular crack growth, as shown in Figure 4.

### 3.2. Microstructural Evolution

**Figure 5:**
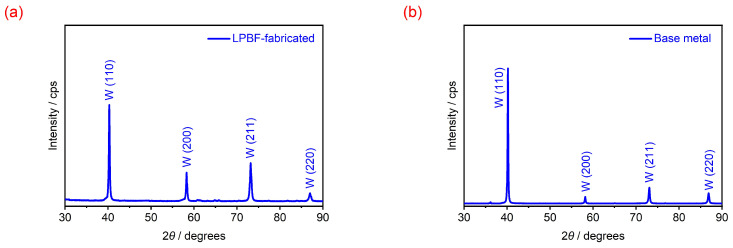
XRD diffraction patterns of the LPBF-fabricated pure W part (**a**) and the corresponding W base metal (**b**).

Figure 5 shows the XRD patterns of the LPBF-fabricated W parts. When combined with the XRD results of Figure 1b, it is clear that the pure W powder, LPBF-fabricated parts, and the corresponding W base metal all exhibited similar diffraction peaks of the B.C.C. crystal structure at (110), (200), (211), and (220) planes, with their peak positions being near identical. This indicates that the LPBF process involved only melting and solidifying the pure W powder, without altering its crystal structure. At the same time, no characteristic peaks of tungsten oxides were found in any of the samples, indicating that W elements were not oxidized during the preparation of W powder or the LPBF of W parts. The diffraction peaks only differed in terms of full width at half maximum (FWHM), with the LPBF sample exhibiting larger values (0.30, 0.35, 0.47, and 0.52) than those of the base metal (0.22, 0.27, 0.36, and 0.38). This is attributed to the fact that the grains of the LPBF samples are finer, while there are residual stresses and defects present. The 2θ positions of the four main peaks remained almost unchanged, being (40.28°, 58.28°, 73.19°, and 87.00°) for the LPBF sample and (40.19°, 58.16°, 73.07°, and 86.87°) for the base metal. According to the Scherrer equation, when the diffraction angles remain the same, a broader FWHM indicates a smaller grain size. Therefore, the observed peak broadening in the LPBF sample can be attributed to grain refinement. During the LPBF process, the large temperature gradient within the molten pool caused a significant difference in solidification rates and solidification shrinkage rates between different positions during solidification, resulting in large internal stresses within the solidified layers. However, due to the presence of a large number of cracks distributed along the grain boundaries within the pure W parts, as shown in Figure 4, the cracks could effectively release the internal stresses generated during LPBF, leading to almost no difference in the 2θ angle positions between the LPBF-fabricated parts (40.28°, 58.28°, 73.19°, and 87.00°) and the W powder (40.29°, 58.24°, 73.18°, and 87.00°).

**Figure 6:**
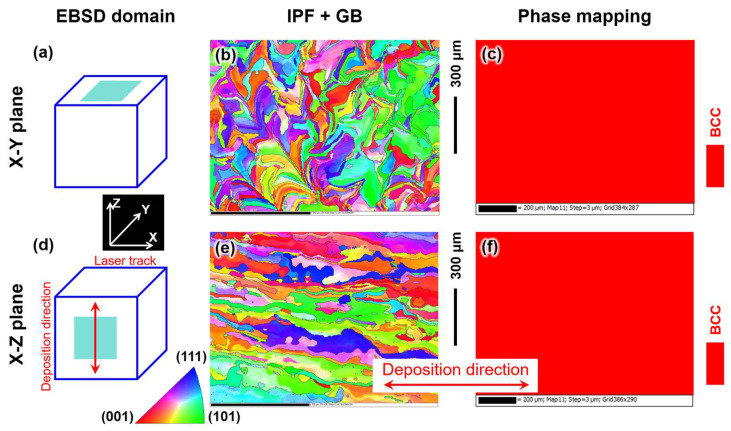
EBSD characterization of the LPBF-fabricated pure W parts: (**a**–**c**) X-Y deposition plane, (**e**,**f**) X-Z deposition plane, (**a**,**d**) schematic graph showing the EBSD domain, (**b**,**e**) IPF map with GB, and (**c**,**f**) phase mapping.

**Figure 7:**
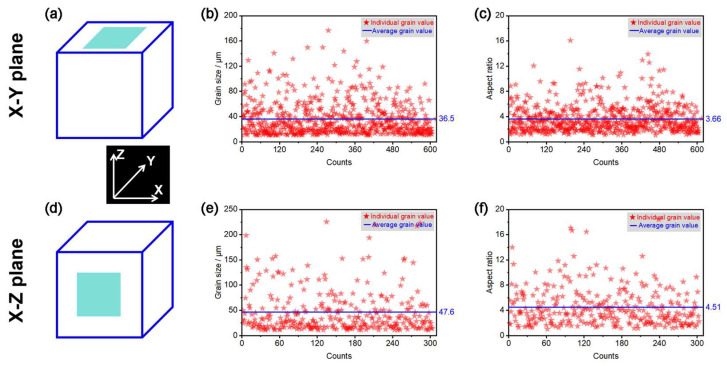
Grain characteristics of the LPBF-fabricated pure W parts: (**a**–**c**) X-Y deposition plane, (**e**,**f**) X-Z deposition plane, (**a**,**d**) schematic graph showing the EBSD domain, (**b**,**e**) grain size, and (**c**,**f**) grain aspect ratio.

Figure 6b,e presents the inverse pole figure (IPF) map along with grain boundary (GB) of the LPBF-fabricated pure W parts within the X-Y and X-Z planes, and the corresponding phase mapping is shown in Figure 6c,f. The grain size and grain aspect ratio in Figure 6 were statistically analyzed, and the results are depicted in Figure 7. Figure 6c,f show that the X-Y and X-Z planes contained only BCC-structured W grains, which is consistent with the XRD results shown in Figure 5a. The average aspect ratio of W grains within the X-Y plane was 3.66. The W grains within the X-Z plane exhibited columnar crystal characteristics, with an average aspect ratio of 4.41. The pure W parts produced by LPBF showed a strong continuity of W columnar crystals growing epitaxially along the vertical height direction, indicating that the as-deposited W grains grew opposite to the heat flow direction. The length of these columnar grains was much greater than the layer thickness of the LPBF-fabricated W parts (layer thickness: 30 μm). The primary cause of columnar grain formation was the heat flow towards the substrate during the LPBF process [38]. Due to extremely high solidification speed (>103 K/s), the layer was completely melted as it scanned, while partially remelting the previously solidified powder layer. The solid–liquid interface then served as a nucleation area for fresh grains. Since the thermal conductivity of the already formed W metal block was greater than that of the W powder, during the layer-by-layer deposition process heat was mainly conducted downward along the deposition direction [39]. The solid–liquid interface advanced along the forming direction, leading to the formation of columnar W grains induced by the temperature gradient. The growth direction of the columnar grains was roughly perpendicular to the substrate.

Comparison of the grain sizes statistics in Figure 7b,e reveals that the W grain size within the X-Y plane (with an average of 36.5 μm) is significantly smaller than that within the X-Z plane (with an average of 47.6 μm). This was primarily due to the use of a rotational scanning strategy within the X-Y deposition plane with an adjacent layer scanning angle of 67°, where the high-energy laser beam had a grain-breaking effect during the remelting of already solidified grains. Due to the temperature gradient distribution along the vertical height direction within the X-Z plane, the liquid molten pool facilitated grain growth from the top of the existing grains. This resulted in the formation of nearly equiaxed grains within the horizontal plane due to the grain-breaking effect. The equiaxed grains were smaller than the epitaxial grains with inherited characteristics within in the vertical plane.

**Figure 8:**
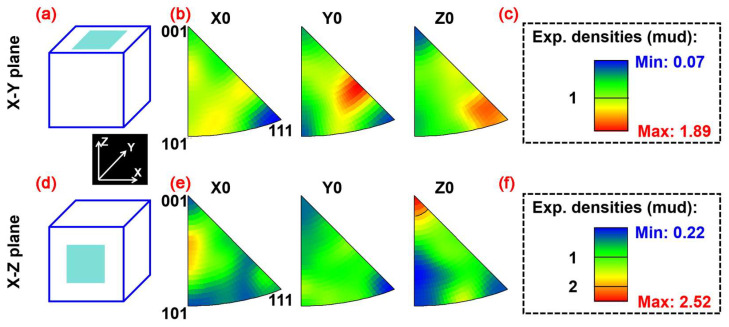
Orientation characteristics of the LPBF-fabricated pure W parts: (**a**–**c**) X-Y deposition plane, (**e**,**f**) X-Z deposition plane, (**a**,**d**) schematic graph showing the EBSD domain, (**b**,**e**) IPF distribution, and (**c**,**f**) phase mapping.

Figure 8 illustrates the grain orientation characteristics of the pure W parts fabricated by LPBF within the X-Y and X-Z deposition planes. Despite the epitaxial growth of W grains along the vertical height direction within the W parts, the grain texture orientation within each deposition plane was relatively weak, with MUD (multiples of uniform density, a parameter employed to quantitatively describe the grain texture intensity) values of 1.89 for the X-Y plane and 2.52 for the X-Z plane.

**Figure 9:**
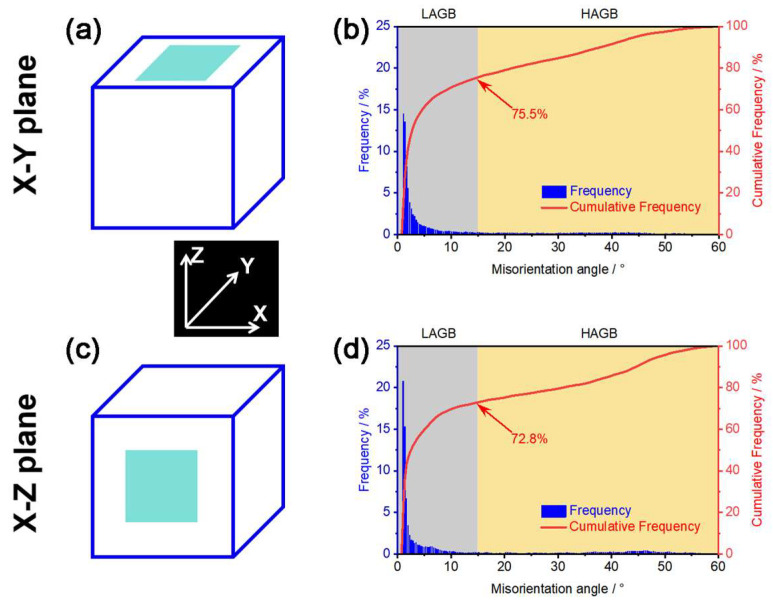
Grain misorientation angle of the LPBF-fabricated pure W parts: (**a**,**b**) X-Y deposition plane, (**c**,**d**) X-Z deposition plane, (**a**,**c**) schematic graph showing the EBSD domain, and (**b**,**d**) frequency and cumulative frequency of misorientation angle values.

Figure 9 depicts the grain misorientation angle of the LPBF-fabricated pure W parts. The distribution of grain orientation angles within the X-Y and X-Z deposition planes was similar, with approximately 75% of the grain boundaries being low-angle grain boundaries (LAGBs). The formation of LAGBs in the W parts was primarily caused by rapid solidification and cooling of the high-temperature small molten pools during the LPBF process, which generated thermal stresses causing localized plastic deformation. The cracks in LPBF-fabricated W parts tend to propagate along high-angle grain boundaries (HAGBs) [40,41]. These cracks were formed during the solidification shrinkage process, originating from the solid–liquid mixture zone at the end of the solidification stage. At this point, the vicinity of the solid-phase zone in the mixture zone contained solidification shrinkage holes, which were prone to local stress concentration, thus facilitating the initiation of cracks. HAGBs typically had lower fracture toughness, which reduced the energy dissipation rate during cracking. Therefore, HAGBs provided a longer, non-restrictive slip path for crack propagation, ultimately leading to the cracks growing along the HAGBs. This means that, unlike HAGBs, LAGBs inhibited crack propagation. Therefore, future research could focus on increasing the number of LAGBs by regulating process parameters or introducing nanoparticles to increase heterogeneous nucleation sites, thereby suppressing cracks in the LPBF-fabricated pure W parts.

### 3.3. Hardness Properties

**Figure 10:**
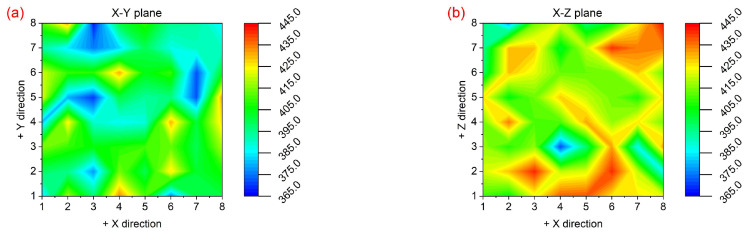
Micro-hardness mapping distribution of the LPBF-fabricated pure W parts: (**a**) the X-Y deposition plane (i.e., horizontal plane) and (**b**) the X-Z deposition plane (i.e., vertical plane).

Figure 10 shows the hardness mapping distribution within both the X-Y horizontal plane and the X-Z vertical plane of the pure W parts produced by LPBF. The hardness fluctuation range is large within both planes. Although the W grains within the X-Y plane are finer and denser than those within the X-Z plane, the large number of cracks distributed along the HAGBs resulted in uneven resistance to external forces within each micro-sized region. Defects such as dislocations and microcracks that formed during the loading process are likely to further move and propagate along the thermal crack paths created during the LPBF process, ultimately leading to a significant difference in the surface hardness between the X-Y and the X-Z planes. The hardness near the cracks was lower than that within the grains due to the poor load-bearing capacity.

**Figure 11:**
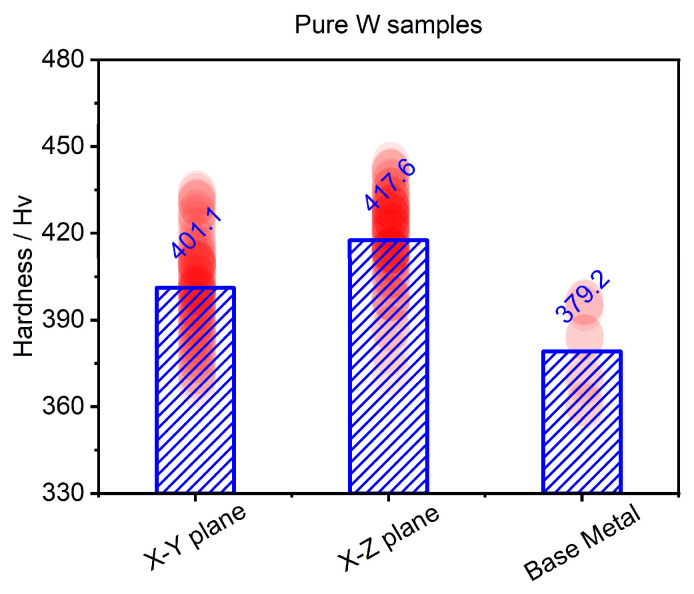
Average values of micro-hardness within the X-Y and X-Z deposition planes extracted from the measured mapping distribution results in Figure 10, along with the base metal. (Histogram: the average value; red circle: the individual value).

Based on the hardness mapping distribution results shown in Figure 10, the average hardness values were statistically analyzed and compared with the hardness performance of the base metal, as shown in Figure 11. The bar chart in Figure 11 represents the average values, and the red circles represent the measured values. When compared to the base metal, the average hardness performance of the LPBF-fabricated pure W within the X-Y and the X-Z planes increased by 22 Hv and 38 Hv, respectively. As shown in Figure 7, Figure 8 and Figure 9, the pure W parts produced by LPBF had denser and finer grains due to rapid melting and solidification, and higher dislocation density and residual stress, all of which significantly enhanced the hardness performance. The average hardness within the X-Z plane was higher than that within the X-Y plane, mainly due to the competing effects of the microstructure and cracks. According to the Hall–Petch relationship, the finer the W grain size, the greater the strengthening effect, and thus the more noticeable improvement in Vickers hardness. As shown in Figure 6 and Figure 7, although the grains within the X-Y plane were relatively refined the crack density inside was higher than that within the X-Z plane, and there was an inverse relationship between crack density and hardness performance. Although cracks were present in both planes, the X-Y plane exhibited a significantly higher crack density, which weakened the potential hardness gain from finer grains and led to an overall lower hardness than that of the X-Z plane.

### 3.4. Wear Performances

**Figure 12:**
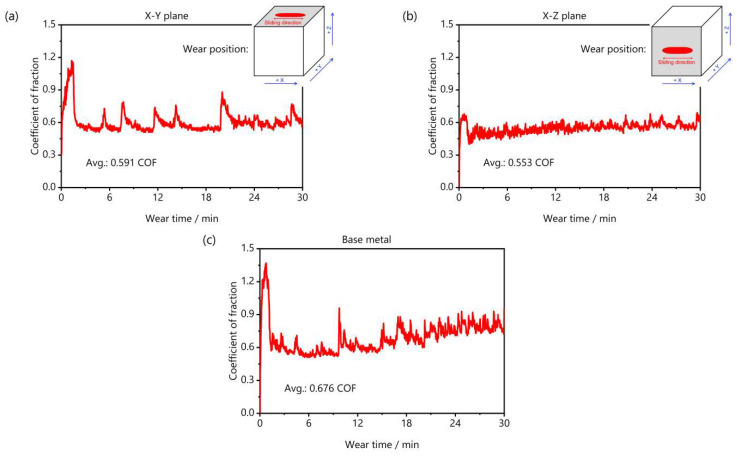
Coefficient of fraction versus wear time curves for LPBF-fabricated W parts and the corresponding W base metal: (**a**) X-Y deposition plane, (**b**) X-Z deposition plane, and (**c**) base metal.

Figure 12 depicts the coefficient of fraction versus wear time curves for LPBF-fabricated W parts and the corresponding base metal. For both the X-Y and X-Z planes of the pure W part and the base metal the friction coefficient initially increased sharply, followed by a gradual rise as the friction time increased, with real-time data showing some fluctuations. This is because, during the initial stage of friction (0–1 min), the sample surface polished with 2000 grit sandpaper was relatively smooth with low surface roughness, resulting in a lower friction coefficient. As the test progressed (1–3 min), the sample surface became damaged, exposing the uneven bare metal and causing the friction coefficient to rapidly increase. The exposed material was highly susceptible to oxidation, which temporarily reduced friction, resulting in a brief decline in the friction coefficient [42]. As friction time further increased (3–30 min), the repair rate of the oxide film was less than the wear rate, causing the oxide film to break and abrasive debris to accumulate on the component surface. This, along with the sample and the friction pair, created three-body friction, resulting in a continued increase in the friction coefficient [43]. As the debris gradually spread over the component surface, causing an imbalance in debris production and overflow, the friction coefficient experienced continuous short-term fluctuations. Figure 12 also shows that the base material has the highest average friction coefficient, followed by the X-Y deposition plane, while the X-Z deposition plane has the lowest. This indicated that the friction performance of each as-deposited plane of the W part was superior to that of the base material, with the X-Z plane showing better friction performance than the X-Y plane, which was consistent with the evolution of hardness performance depicted in Figure 11. Similarly to hardness performance, improved wear resistance within the X-Y plane was caused by competition between the internal defects (Figure 4) and microstructural characteristics (Figure 6).

**Figure 13:**
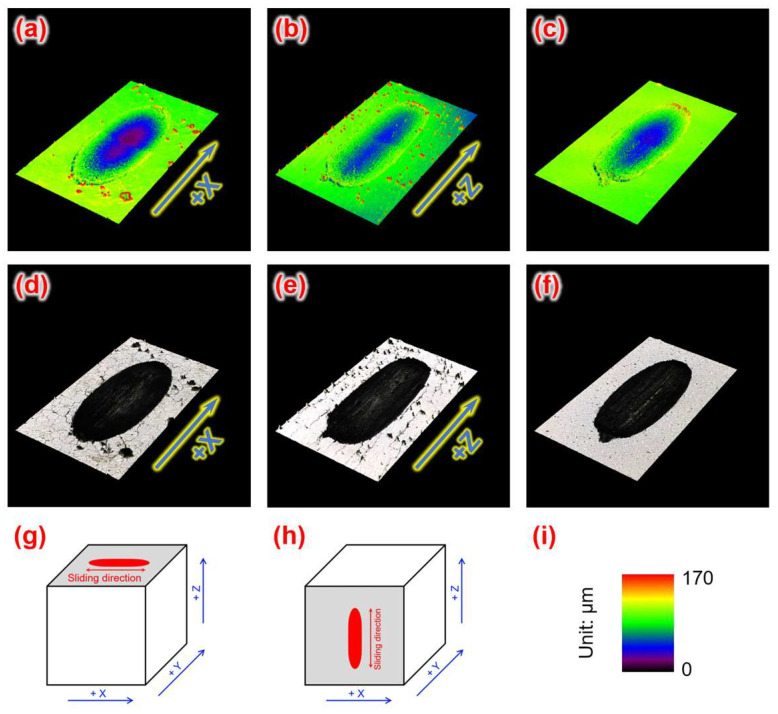
Three-dimensional macro-morphology of wear tracks of W samples: (**a**,**d**,**g**) X-Y deposition plane of LPBF-fabricated W part, (**b**,**e**,**h**) X-Z deposition plane of LPBF-fabricated W part, (**c**,**f**) W base metal, (**a**–**c**) rainbow morphology chart, (**d**–**f**) physical morphology chart, (**g**,**h**) schematic graph showing wear domain, and (**i**) legend of rainbow chart.

**Figure 14:**
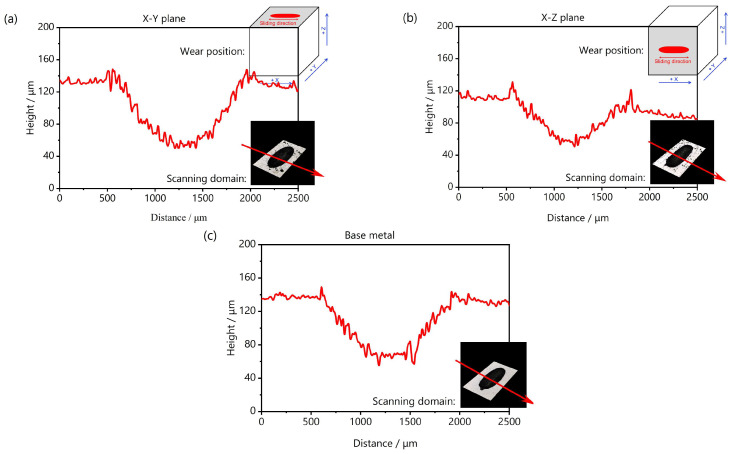
Surface profile along the middle position of the side of wear zone: (**a**) the X-Y deposition plane of LPBF-fabricated W part, (**b**) the X-Z deposition plane of LPBF-fabricated W part, and (**c**) the W base metal.

Figure 13 shows the macroscopic LSCM morphology of the wear scars. The wear track contours of the three samples are oval-shaped when viewed from the top. Material displacement occurred along the sides of the wear scars due to the plowing effect of dry friction, with the depth gradually increasing from the sides to the center. To better compare the wear behaviors of different samples a profile line was drawn from the middle of the wear scar, and the results are shown in Figure 14. The width and depth of the wear scar within the X-Z plane of the LPBF-fabricated W part were smaller than those within the X-Y plane, indicating that the X-Z plane had better wear-resistant characteristics. This explains why the X-Z plane had a lower friction coefficient than the X-Y plane.

**Figure 15:**
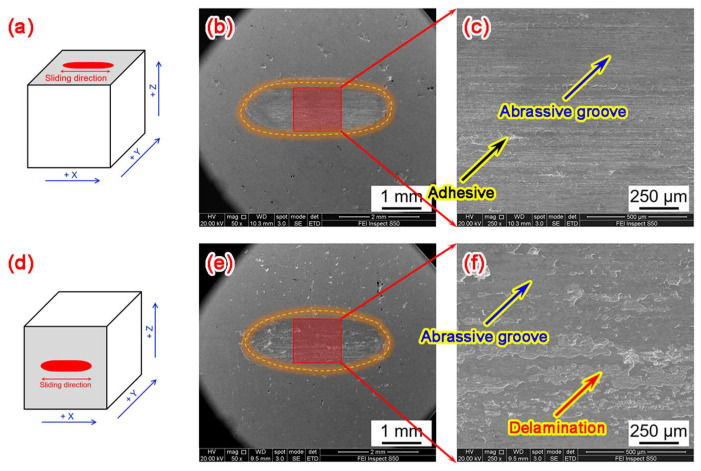
SEM images of wear morphology of LPBF-fabricated pure W components: (**a**–**c**) X-Y deposition plane, (**d**–**f**) X-Z deposition plane.

To investigate the wear-resistant mechanism of the LPBF-fabricated W part, SEM was used to observe the morphology of the wear scars, and the results are in Figure 15. Figure 15a–c of the X-Y deposition plane indicates that the surface was clearly characterized by abrasive grooves parallel to the wear direction along with a small number of adhesive points, indicating that its wear failure behavior was abrasive wear with slight adhesive wear. As shown in Figure 15d–f of the X-Z deposition plane, the surface morphology of the wear scar was significantly different from that of the X-Y plane. The surface of the wear scar was composed of abrasive grooves and delamination pits that were aligned along the wear direction. The formation of delamination pits was attributed to the separation of W material from the surface caused by friction between the friction pair and the metal, leaving hard particles on the surface. These hard particles continuously impacted the surface micro-protrusions under cyclic tensile and compressive stresses and eventually detached from the substrate under the action of shear stress to form abrasive particles. This results in a three-body friction of the substrate–abrasive particles–friction pair, leading to the formation of delamination pits. The wear failure behavior of the X-Z plane was a comprehensive wear mechanism characterized by both delamination wear and abrasive wear.

By comparing the wear scar morphologies in Figure 15, it is clear that the wear behavior of the LPBF-fabricated W part was transformed from a comprehensive wear mechanism involving delamination wear and abrasive wear within the X-Z plane to an abrasive wear mechanism with slight adhesive wear within the X-Y plane. As shown in Figure 4 and Figure 6, the X-Z plane was primarily composed of coarse columnar grains, accompanied by numerous elongated cracks distributed along HAGBs. Under prolonged cyclic compressive and tensile stresses during wear, these cracks tend to initiate and propagate more readily, eventually leading to delamination and material spalling. Although the X-Y plane exhibited a fine equiaxed grain structure with a smaller average grain size compared to the X-Z plane (Figure 7), a higher density of reticulated microcracks was observed along the horizontal plane. These defects compromised the mechanical integrity and resulted in a reduction in hardness, thereby leading to the occurrence of slight adhesive wear during the wear process. As a result, the wear scars on the X-Y plane revealed a higher wear volume (Figure 14), characterized by more pronounced abrasive grooves and the presence of a few adhesive points, thereby indicating inferior wear resistance compared to the X-Z plane.

## 4. Conclusions

This work employed LPBF technology to manufacture refractory W parts and explored the internal defects, microstructural characteristics, Vickers hardness, and wear resistance in both the horizontal and vertical planes to evaluate their anisotropic behavior. By correlating these properties with the build orientation, this study offers new insights into the anisotropic behavior of LPBF W parts. The key findings are concluded as follows:

(1) The top and side surfaces of the LPBF-fabricated W parts exhibited excellent deposition quality, with only a small number of pores and cracks observed. Additionally, the surface roughness was significantly reduced when compared to the size of the original powder particles. However, an array of elongated cracks aligned with the HAGBs was observed within the part, primarily owing to the grain boundaries providing extended and unobstructed slip paths for crack propagation.
(2) The steep temperature gradient during the LPBF process has led to the formation of columnar crystals, which grew along the vertical direction. The grain size within the horizontal plane was finer than that within the vertical plane, and the intensity of grain orientation within both deposition planes was relatively low. The proportion of LAGBs reached 75% due to high thermal stresses generated by rapid solidification and cooling, causing local plastic deformation within the grains.
(3) The distribution of cracks resulted in significant variations in hardness properties across the micro-regions within the horizontal and vertical planes. The hardness performance was a result of the competition between the grains and cracks. The numerous cracks distributed along the HAGBs within the horizontal plane significantly weakened the fine-grained strengthening effect, leading to a lower average hardness performance within the horizontal plane compared to the vertical plane.
(4) Similarly to the hardness performance, wear resistance within the vertical plane was superior to that within in the horizontal plane. The surface of the wear scars within the vertical plane are composed of abrasive grooves and delamination pits, whereas within the horizontal plane there are clear observations of abrasive grooves accompanied by a small number of adhesive points. The wear behavior transformed from a comprehensive wear mechanism involving delamination wear and abrasive wear within the vertical plane to an abrasive wear mechanism with slight adhesive wear within in the horizontal plane.

## Data Availability

The original contributions presented in this study are included in the article. Further inquiries can be directed to the corresponding authors.
